# The key reasons for dropout in Slovenian music schools – a qualitative study

**DOI:** 10.3389/fpsyg.2024.1385840

**Published:** 2024-05-30

**Authors:** Ana Kavčič Pucihar, Katarina Habe, Branka Rotar Pance, Maruša Laure

**Affiliations:** ^1^Academy of Music, University of Ljubljana, Ljubljana, Slovenia; ^2^Faculty of Education, University of Maribor, Maribor, Slovenia

**Keywords:** music schools, instrumental music education, dropout, music students, parents, instrumental music and theory teachers, music school principals

## Abstract

Music education often struggles to sustain students’ long-term commitment, with many perceiving lessons as frustrating or unengaging, leading to discontinuation. To address this gap, our study aimed to elucidate the primary reasons for dropout from the perspectives of various stakeholders, including students, parents, teachers, and principals. Drawing upon the self-determination theory, our research comprehensively investigated external and internal factors contributing to dropout. Among external factors, competing extracurricular commitments, music theory and solfége lessons, and teacher’s approach emerge as the most prominent. Among internal factors, our findings highlighted the critical role of autonomy, competency, and relatedness in shaping students’ decisions to continue or discontinue music education. Inadequate teacher-student relationships, limited peer interactions, and uninspiring classroom atmospheres significantly impacted dropout. Moreover, challenges in the music school curriculum, such as difficulties with music theory and solfège, resource limitations, and excessive workloads, emerged as prominent barriers to student engagement. By addressing these multifaceted issues, our study underscores the importance of fostering supportive environments that cater to individual needs and interests, ultimately enhancing the overall music education experience and reducing dropout rates. This research represents the first systematic empirical study in Slovenian music education, laying the groundwork for future quantitative investigations to advance education practices in Slovenia.

## Introduction

Teaching and learning music have been recognized to serve various human needs. Blackwell & McPherson (2022, p. 72) argue that “over 95% of the population can benefit from systematic music education, and with sufficient practice and systematic training, can even develop their musical potential to a professional level.” Reimer (1999) describes teaching and learning music as a way to improve one’s ability to gain meaningful and gratifying musical experiences. “Learning a musical instrument can be one of the most enjoyable and rewarding hobbies or pastimes a child can pursue. However, it can also be one of the most frustrating” (McPherson et al., 2015, p. 418). An »alarmingly high« proportion of music students who start to learn a musical instrument subsequently give up (North et al., 2000, p. 270). The first significant surge of dropout comes at the age of 11 (Evans, 2009) and between ages 15 and 17 (Ruth and Müllensiefen, 2021). Williams (2002) suggests that the difficulty of keeping one’s realistic perspective of one’s self-image during adolescence contributes to one’s decisions to continue or discontinue music study. Many music students gave up before achieving even basic proficiency, feeling dissatisfied with their learning experience and disillusioned with musical activities (Evans, 2009). In many cases, students enthusiastically start their lessons but discontinue them before attaining the skill level necessary for musical independence and satisfaction (Costa-Giomi et al., 2005).

Children, youth, and adults participate in music education in formal and informal learning practices (Green, 2017). The array of informal learning practices in music education has been growing in Slovenia. However, this research is focused on the Slovenian formal music education system, specifically for students between seven and 15 years of age. According to the report of the Republic of Slovenia Statistical Office (SURS (2014/2015), 2015), 20, 630 pupils were enrolled in the instrumental music education program in Slovenian music schools. In the previous school year (2013/2014), only 2, 788 pupils successfully finished the 1st or 2nd tier of their instrumental musical education in Slovenian music schools (SURS (2014/2015), n.d.). This steep dropout has yet to be researched in the context of the Slovenian music school system.

### Slovenian music education system

A brief outline of the structure and history of the music education (instrumental and vocal) system in Slovenian schools is provided to explain the context to international readers:

The state of Slovenia provides two main types of music education for children and teenagers between the ages of 6 and 19: classroom music education and instrumental and vocal music education (Eurydice, 2024). Classroom music education occurs in elementary schools (children between the ages of 6 and 15) and several high school programs (students between the ages of 15 and 19).

At the elementary level, instrumental and vocal music education occurs in specialized music schools organized in a network of elementary music schools across Slovenia. Five music high schools are at the intermediate level, and one Academy of Music at the University of Ljubljana (AEC Music, 2017).

Slovenian music schools are integral to the European Music School Union (Rotar Pance, 2019; EMU, 2022; Hahn et al., 2024), representing a system with a 200-year-old tradition. This system, evolving significantly after Slovenian independence in 1991 (Rotar Pance, 2012), includes state-run music schools and government-approved private music schools, amounting to 54 public and 17 private institutions (Music Institutions Register, 2024). Slovenian music schools offer programs in music and dance education. In the following section, we focus on instrumental music education programs.

There are 27,161 music school students in the school year 2023/2024, which is 12.61% of the Slovenian school population. Most (84%) are elementary school students aged 7 to 15. There are 22,027 students enrolled in the MUSIC program – instrumental music education or singing (Ministry of Education, 2024).

The Music Schools Act of 2000, amended in 2006, and the related implementing regulation provide the legal framework for this education system, setting forth goals such as: “talent identification, personality development, improving the overall level of the education, and establishing a base of musical knowledge and experience to enable participation in amateur instrumental ensembles, orchestras, choirs, or dance groups” (Music Schools Act, 2000/2006; par. 2). Among other goals, it also prescribes supporting students’ personal development per their abilities and the development principles.

The journey of individual instrumental education in Slovenia typically commences at the age of seven, although this may vary depending on the instrument. To enroll in instrumental classes, students must first pass a musical ability entrance exam, ensuring a certain level of proficiency. The curriculum is structured into two tiers: the 1st tier of instrumental music education spans 6 years, followed by the 2nd tier, which lasts 2 years.

The curriculum includes individual lessons on the chosen instrument, classroom theory or solfège classes, and a gradual introduction to ensemble playing and orchestra. In the first 3 years of study, the curriculum consists of two 30-min weekly individual lessons on the chosen instrument and one 45–60-min group theory or solfège lesson. In the 4th year, students of most instruments start to play in some form of ensemble setting (wind band, string orchestra, symphony orchestra, or choir singing). Over the course of 8 years, students are systematically assessed and graded in all music school subjects, including instrumental performance, theory, and ensemble participation, providing a comprehensive learning experience. The curricula are structured around specific learning outcomes as well as progressive learning outcomes. Such a structure should enable music teachers to provide the required differentiation for each student – especially in one-to-one individual instrumental instruction. However, there is no precise data on how this is carried out in everyday music school practice. The necessity of passing annual exams, except in the first grade, shows a systematic yet rigorous approach toward providing a high-quality foundation for instrumental music education.

Although the Slovenian music education system is very specific, there are some similarities with various international types of music education. In Slovenian music schools, children between the ages of 7 and 15 have instrumental music lessons in the form of individual instrumental instruction, internationally known as private studio instruction or one-to-one tuition. The theory and solfège lessons are held similarly to what is internationally known as music theory classes at the higher education level (university), while their ensemble lessons are similar to wind band or choir rehearsals held at schools.

Research on dropout in group and individual musical instrument instruction is presented from now on, as students in Slovenian music schools participate in both types of instrumental education. The age of participants in the following literature review corresponds with the age of Slovenian music school students – 7 to 15 years unless stated otherwise.

While there is a well-established body of literature on dropout in school band and orchestra programs, more research is needed in the context of private studio musical instrument individual teaching. Existing research on reasons for dropping out of instrumental music education discovers intertwining external and internal factors.

### Research on dropout in school band and orchestra programs

Research on dropout in school band and orchestra programs finds the following main external dropout factors: other competing interests and commitments (Cook, 2013; Hurley, 2021; Hash, 2022), logistical issues, including scheduling (Kinney, 2010; Busch et al., 2012; Cook, 2013; Hash, 2022) and issues associated with students’ social environment. These include lack of support among peers (Cook, 2013), lack of parental support (Cook, 2013), inadequate parental or teacher support (Pitts et al., 2000), and family structure. There are higher levels of dropout among students from single-parent families (Kinney, 2010), and higher levels of mothers’ expressed concerns about practice reflected in higher levels of their children dropping out from instrumental training (McPherson and Davidson, 2002). Socioeconomic status – the lower the SES, the higher the dropout rate is strongly associated with the dropout phenomenon in group music instruction (Corenblum and Marshall, 1998; Albert, 2006; Kinney, 2010; Busch et al., 2012).

Internal contributing factors toward dropout include academic achievement – students who struggle academically may be more prone to drop out (Gamin, 2005; Kinney, 2010); students’ attitudes toward their musicianship – if students do not find personal satisfaction in instrumental music, they are more likely to drop out (Hash, 2022); unwillingness to spend time for instrument practice (Gamin, 2005; Cook, 2013) and loss of motivation (Busch et al., 2012).

Krause et al. (2020) examined reasons for dropping out of participation in musical activities among 190 Australian residents aged 17–75 years. In retrospect, the participants provided answers that can be placed in both previously presented categories: “access and opportunity” and “obligations” correspond with the external factors category, while “activity experience” and “difficulty with practice” correspond with the internal factors category.

Gerelus et al. (2017) point out that more than research focused on dropout with band and orchestra students may be needed to generalize findings to individual studio instruction, as later has its unique challenges, different from group instrumental teaching. Gerelus et al. (2017, p. 29) name factors such as »difficult solo repertoire, close teacher relationship, lack of social group aspect and a large extracurricular time commitment« as making private individual lessons different from group music teaching.

### Research on dropout in individual instrumental instruction

Contexts of research on individual instrumental instruction slightly differ in each case. Therefore, each is presented in more detail. Costa-Giomi (2004) and Costa-Giomi et al. (2005) found behavioral differences among persisting and dropout piano students. Dropout students were less likely to have siblings, missed more lessons, practiced less, completed less piano homework, and achieved lower scores on piano exams than continuing students. They achieved less from the beginning of their studies than their continuing peers. Costa-Giomi (2004) identified lowered motivation and diminished achievement as early predictors of dropout behavior in piano students. In a subsequent study, Costa-Giomi et al. (2005) found that dropout students elicit more verbal cues from the teacher but get fewer praises from the piano teacher as they accomplish fewer assigned goals. Behavioral differences may help identify late but not early dropouts (Costa-Giomi et al., 2005).

King (2016) identified the primary reasons for dropping out of piano studio instruction. Predictors of musical ability, musical achievement, practice habits, and long-term commitment could accurately predict dropout but did not always impact motivation. Dropout students began lessons later in childhood, had less overall musical ability, weaker practice habits, and were progressing far more slowly than the continuing students. The main reasons for stopping lessons included lack of practice, preferring other instruments, and losing interest. The only predictor that impacted motivation was the quality of parental involvement. King (2016) reached an important conclusion about the connection between motivation and dropout; she found that dropout piano students were significantly less autonomously motivated than their persisting peers.

Gerelus et al. (2017) researched the role of *expertise* (musical ability, academic achievement, and musical achievement) and *environment* (social and educational status, gender differences, parental involvement, and home culture) in students’ decisions to drop out of piano lessons. Dropout students reached significantly lower playing levels, despite taking lessons longer, had much higher instances of stay-at-home mothers, and fewer academic or professional mothers; in general, dropout group students’ mothers were overall less educated than mothers of persisting students. Gerelus et al. (2017) describe dropout group parents’ behavior as being overbearing and contributing to student dropout.

Gerelus et al. (2020) found significant differences in types of motivation between dropout and persisting piano students. Dropout students demonstrated less autonomous motivation and stronger amotivation. They started to play the piano later in their childhood than the persisting students and practiced less, although practicing was not necessarily related to motivation. The authors state that dropout students may have lacked competency, relatedness, and autonomy, which resulted in feelings of amotivation. They define a lack of competency as connected with dropping out. As in King’s (2016) research, Gerelus et al. (2020) found parental involvement related to dropping out – parents sitting in lessons were negatively correlated with autonomous motivation, as parents may have overstepped their boundaries to interfere with the lesson setting. The level of autonomous motivation of dropout students was insufficient to sustain further music study. The authors state that the decision to drop out is connected to a lack of autonomous motivation.

Contributing external and internal factors to dropout are quite similar to those in the school band or orchestra settings, but there are some differences: in the internal factors category, piano dropout students had weaker long-term commitment, lower overall musical accomplishment, and musical ability. In the external factors category, they started playing piano later in their life, missed more lessons, were less likely to have siblings, and had higher instances of stay-at-home mothers who were overall less educated than mothers of continuing students (Costa-Giomi, 2004; King, 2016; Gerelus et al., 2017, 2020).

Loss of motivation is a recurring factor in relation to dropping out in both types of instrumental music education. Therefore, it requires further examination.

### Motivation in music education

Motivation attributes between 12 and 27% of music achievement (Asmus, 2021), yet there is »a limited number of studies on children’s motivation in the context of learning a musical instrument« (Oliveira et al., 2021, p. 105). “Providing an autonomy-supportive, musically stimulating, and encouraging environment may prove more important than any predispositions to musical learning” (Blackwell and McPherson, 2022, p. 75).

Pitts et al. (2000) investigated the motivations and behavior of young instrumentalists in their first 20 months of learning. They compared motivation in students who ceased lessons and the ones who persevered. The complexity of musical learning is clearly shown in their research, as they describe the interplay of motivation, practice strategies, environment, and personality acting in proportions that authors find difficult to separate. Pitts et al. (2000) found that even the most motivated children had periods of self-doubt and required support from the teacher and parents. Authors suggest that students who lose motivation lack self-efficacy or external support and are especially susceptible to other negative influences. They point out that empathetic parental and teacher support is essential to progress, as is effective practice; they claim children ought to be taught and implemented (Pitts et al., 2000).

McPherson et al. (2012, p. 59) define »*demotive factors«* in the instrumental learning context: »serious short-comings about the quality of practice sessions, lack of parental support, significant antagonism around practice sites, boredom, an absence of personal engagement, limited learning autonomy over nearly all areas of learning, restrictive forms of music making and learning (i.e., the dominance of performance from notation and absence of other forms of performance like playing by ear and improvising), and, for many, very limited progress in terms of musical skill development in both instrumental/technical and notational/literacy areas« (McPherson et al., 2012, p. 59).

Inadequate teaching materials (Asmus, 2021), social comparison, normative grading criteria, public evaluation forms, ability self-assessment, and competitive musical environment (West, 2013) decrease motivation. McPherson et al. (2016) recognize many teaching practices undermining student autonomy and motivation in the classical master-apprentice lesson setting.

### Self-determination theory as a theoretical framework for investigating music(de)motivation

Self-determination theory (Ryan and Deci, 2002) has received wide recognition in the context of instrumental music education research (Evans et al., 2012; Evans, 2015; Freer and Evans, 2018; Gerelus et al., 2020; Lee and Leung, 2020; Oliveira et al., 2021; Shaheen, 2022). Self-determination theory explains why an individual is motivated to engage in an activity or to stop engaging in it according to the satisfaction of three psychological needs: competence, relatedness, and autonomy. The need to feel competent is the desire to feel adequate and successful in acquiring and performing skills. The need for coherence is the desire to feel socially connected and integrated. The need for autonomy is the desire to feel self-determination and control over one’s activity (Ryan and Deci, 2002).

From the perspective of self-determination theory (Deci and Ryan, 1985), children’s motivational decline and eventual dropout occur when their psychological needs for autonomy, competence, and relatedness in their musical learning are »being thwarted« (Evans et al., 2012, p. 17). Evans (2015) suggests that rather than questioning how to motivate students, parents, and teachers should strive to create social environments in which their “students are more likely to generate their own interest, enjoyment, and motivation so that they can identify the value of musical practice, integrate it with their sense of self” (Evans, 2015, p. 78).

### Music teachers

There is a duality of dimensions in music teachers, as they need to be both musicians and teachers. They must develop a professional identity and professional activity. During their studies, future music teachers are trained in their musical instrument mastery as well as in the field of pedagogy. The development of both identities is not parallel. Through the educational process of future music teachers, more emphasis is put on developing the identity of a musician/performer compared to the identity of a music teacher (Rotar Pance, 2019).

Blackwell & McPherson (2022, p. 72) outline 12 general principles for those »who provide instrumental and vocal lessons to children: » (1) everyone can benefit from music education, (2) start early, (3) immerse the child in other aspects of music, beyond learning to perform, (4) allow choice when selecting and choosing to change instruments, (5) consider the developmental appropriateness of the learning environment, (6) motivation is the key to success, (7) understand how students learn, (8) make the learning journey »visible« in order to foster a sense of musical identity, (9) do not use tests of music aptitude to determine who learns music, (10) design learning to minimize biases and stereotypes, (11) focus on the love of music and avoid external rewards, pressures, and controls; (12) develop a healthy state of mind through support, love, and encouragement».

As the teacher’s role in individual musical instrument instruction dropout has yet to be researched in more detail, the existing research does provide some answers: music teachers point to two significant dropout factors in students: unwillingness to spend time practicing and poor academic performance (Gamin, 2005). Williams (2002) points to the complexity of students’ motivation to continue or discontinue musical studies. Individual instrument teachers can detect early signs of students’ motivation decreasing and intervene accordingly (Williams, 2002). He indicates that some parents allow their children to take full responsibility for their learning, while some students hold their teachers responsible for the outcomes of their lessons.

Davidson et al. (1998) researched music teachers’ characteristics and the young instrumentalists’ progress. Their study included 257 young people between the ages of eight and 18 who had received instruction on at least one musical instrument. They divided the participants into 5 groups. In Groups 1–4, there were active musicians – from highly successful to amateur. In Group 5, young ex-musicians are children who dropped out of music lessons. The students in Group 5 rated their instrumental teachers with »the least positive ratings« (Davidson et al., 1998, p. 149) in the following characteristics: friendly – unfriendly; relaxed to tense; chatty to quiet; encouraging to not encouraging; pusy to unpushy; good teacher to bad teacher and good player to bad player. The dropout students rated their last teachers lower in encouragement than all the other groups. They also rated their last teachers as significantly worse than any other group.

Hash (2022, p. 13) claims that »exactly how students’ positive or negative feelings toward their instrumental music teachers affect retention remains unclear« and suggests that »almost all decisions made by instrumental teachers have the potential to influence student retention« (Hash, 2022, p. 39).

### Dropout in the extracurricular activities

Similar to music activities, sports are popular after-school activities in many ways. In both fields, the dropout phenomenon of early cessation of activity is common. Roček et al. (2021, p. 72) state that “we still face a massive dropout of children from the sports, which is not replaced with an adequate alternative physical activity.” Fraser-Thomas et al. (2016) and Woods and Butler (2021) state that 50–70% of youth participate in organized sports activities in Westernized nations. Still, around 35% of participants leave youth sports programs annually, and by the age of 13, there is an estimate that 70% of youth leave sports activities. This way, they are losing out on developmental and health benefits (Battaglia et al., 2024). Crane and Temple (2015) find five major areas contributing to dropout in sports among children and youth: lack of enjoyment, perceptions of competence, social pressures, competing priorities, and physical factors (maturation and injuries). Back et al. (2022) found that intrapersonal constructs related to motivation and sports experience had the strongest relationship with dropout.

Roček et al. (2021, p. 72) state that the consequences of dropping out of sports activities bring a range of personal, health, and social problems. While the impacts of instrumental music education dropout might not be as detrimental, Pitts and Robinson (2016) underline the important role of music education in laying the foundations for lifelong participation and providing all children with experience and understanding of making music.

In McPherson et al. (2012, p. 56) study in Australia, “three-quarters of the 104 surveyed adults had given up musical instrument playing, and negative views of their instrumental learning experiences were widely reported.” There is a considerable variety in instrumental music education praxis worldwide. Therefore, dropout numbers are generally hard to obtain. Moreover, they cannot be easily compared. The existing data across countries, albeit at different points in time, show that dropout in instrumental music education is steep:

According to the AMA (2001), about 25% of Australian students drop out by age 12, with another 25% discontinuing by age 15, citing boredom, loss of interest, and little motivation as reasons for dropping out (StGeorge, 2006); in Serbia, 22% of students between the ages 7 and 12 leave their instrumental tuition in music schools within the first 2 years (Bogunović, 2010). The exact data on the percentage of students who drop out of Slovenian music schools in Slovenia has not been purposefully collected and cannot be obtained. However, the Republic of Slovenia Statistical Office (SURS (2014/2015), n.d.) reports the difference between the number of students enrolled in the 1st classes of the Slovenian music school MUSIC program (20, 630) and the number of students who successfully finished the MUSIC program in the previous school year (2, 788). These two numbers would suggest an 86.5% dropout from Slovenian music schools. However, this percentage must be tentatively interpreted, as there is no available data on the number of students enrolled in the 1st class in the 2008/2009 school year to obtain within one music school generation dropout percentage. Moreover, the number of available places in the 1st classes slightly varies yearly. From the available data we can conclude that the drop out is steep.

### Aims of the study

The presented existing research provides valuable insights into the interplay of internal and external factors contributing to dropout in instrumental music education worldwide. The research on youth sports activities provides further understanding. However, no available data illuminates the interplay of internal and external factors in a systematic music education system such as Slovenian. No similar research has been done in Slovenia. The following study fills the gap in understanding contributing factors leading to dropout in Slovenian music schools.

The main aim of this study is to explore the contributing factors for dropout and motivational decline of music students aged 7 to 15 in Slovenian music schools. To systematically investigate these factors, the following general research question was formed: What are the perceived factors contributing to dropout in Slovenian elementary music schools, according to dropout students, their parents, music instrument teachers, music theory teachers, and school principals?

## Method

A qualitative content analysis, as outlined by Dey (2005), was selected as the methodology to systematically investigate the factors influencing dropout rates among students in Slovenian elementary music schools. The analysis was conducted on data gathered from semi-structured focus groups, enabling the systematic examination of perceived dropout factors and identifying emergent themes.

### Sample

The research was structured around five focus groups, consisting of (1) dropout music students who recently discontinued public music school, (2) the parents of these students, (3) instrument teachers, (4) music theory teachers, and (5) principals of elementary music school. A purposive sampling method (Patton, 1980) incorporating both quota sampling (which involves selecting participants to ensure the sample reflects certain characteristics of the broader population) based on predetermined inclusion and exclusion criteria and snowball sampling (which involves existing study participants referring future participants from their network, useful in accessing hard-to-reach populations) techniques, facilitated the selection of the participants. This approach ensured a diverse sample, capturing a broad spectrum of perspectives on the issue of student dropout. Information regarding the socio-demographic composition of the sample, such as age, gender, and musical instrument of study, was collected via a structured questionnaire.

*Students (N = 6)*

Inclusion criteria: (i) aged between 7 and 14 years, (ii) dropped out of a public music school in the last 2 years.

Exclusion criteria: lack of parental consent.

*Parents (N = 6)*

Inclusion criteria: parents of the children included in the study.

*Instrument teachers (N = 6)*

Inclusion criteria: (i) instrument teacher at a public music school, (ii) at least 1 year of experience in teaching an instrument at a public music school.

Exclusion criteria: (i) only one teacher per music school could be included, (i) only one teacher of the same instrument could be included in the focus group.

*Music theory teachers (N = 6)*

Inclusion criteria: music theory teacher at a public music school, at least 1 year of experience in teaching music theory at a public music school.

Exclusion criteria: only one music theory teacher per music school could be included.

*Principals (N = 5)*

Inclusion criteria: principal of a public music school.

The study included participants with diverse characteristics, including different gender, age, and, for students and instrument teachers, the variety of instruments played.

### Procedures

Data collection was conducted in compliance with ethical standards, including the Helsinki Declaration and the Personal Data Protection Act. The research received ethical approval from the University of Maribor’s Faculty of Arts Ethics Committee. Participation was voluntary, with participants being free to withdraw at any point, and informed consent was obtained through signed forms. To ensure anonymity, all identifiable information was removed from the data. Participants were assigned pseudonyms in all research documentation and analysis. Identifiable information, such as names or specific locations, was omitted or generalized in the transcription process.

The data analysis was conducted using a structured qualitative content analysis approach. Initially, audio recordings were transcribed, organized, and coded to identify statements pertinent to the research problem. An initial list of 52 codes was generated, reflecting a diverse range of responses related to the research question. Through a systematic process of refinement involving multiple rounds of analysis and discussion among the research team, these codes were examined for conceptual similarity and thematic relevance. This iterative process led to consolidating the initial codes into 9 distinct themes. A combination of inductive and deductive coding strategies was used. Theme identification was a two-step process: first, an open coding phase where themes were identified based on the data itself, and second, a reflective phase where these themes were considered in relation to prior theoretical constructs. The analysis was conducted in Atlas.ti and NVivo. The primary analyst conducted the coding and theme development, with periodic consultations with co-authors to validate the coding scheme and the interpretation of themes.

## Results

The analysis of focus group data yielded nine themes categorizing the perceived factors contributing to student dropout in Slovenian music schools. These themes are divided into four internal and five external factors, providing a comprehensive overview of the influences on students’ decisions to discontinue their music education. For detailed descriptions of each theme and associated codes, refer to Tables 1, 2 (Internal Factors) and Table 3 (External Factors).

**Table 1:** Sample characteristics.

Participant	Age	Gender	Instrument	Relationship
Student 1	10	Female	Piano	Child of parent 1
Student 2	11	Male	Percussion	Child of parent 2
Student 3	12	Female	Flute	Child of parent 3
Student 4	10	Female	Flute	Child of parent 4
Student 5	12	Female	Oboe	Child of parent 5
Student 6	13	Male	French horn	Child of parent 6
Parent 1	35	Female	/	Mother of student 1
Parent 2	48	Female	/	Mother of student 2
Parent 3	47	Female	/	Mother of student 3
Parent 4	39	Female	/	Mother of student 4
Parent 5	49	Female	/	Mother of student 5
Parent 6	61	Male	/	Father of student 6
Instrument teacher 1	49	Male	Trombone	/
Instrument teacher 2	49	Male	Violin	/
Instrument teacher 3	46	Female	Accordion	/
Instrument teacher 4	43	Female	Piano	/
Instrument teacher 5	31	Male	Percussion	/
Music theory teacher 1	54	Female	/	/
Music theory teacher 2	36	Male	/	/
Music theory teacher 3	48	Male	/	/
Music theory teacher 4	30	Female	/	/
Music theory teacher 5	48	Female	/	/
Music school principal 1	58	Male	/	/
Music school principal 2	46	Female	/	/
Music school principal 3	51	Female	/	/
Music school principal 4	53	Male	/	/
Music school principal 5	55	Female	/	/

**Table 2:** Internal factors.

*Theme*	*Category*	*Code (N of occurrences)*
Autonomy	Limited repertoire choice autonomy	Reading music (4)
		Teachers’ (lack of) flexibility (3)
		Musical pieces from popular music genres (3)
		Repertoire for technique development (3)
	Parent-initiated instrument learning	Learning music for parents’ sake (2)
Competency	Underdeveloped musical abilities and skills	More demanding repertoire in the higher classes (6)
		Underdeveloped musical abilities (2)
		Comparison with siblings (1)
	Competitive setback and diminished competence perception	Reading music (4)
		Musical exams (2)
		Disappointment after the competition (2)
		Learning music requires a lot of effort to achieve results (2)
Relatedness	Poor teacher-student relationship	Flexibility of teachers, lack of patience (7)
		Poor teacher-student relationship (5)
		Teacher-student relationship quality (2)
	Limited peer relations	Playing in musical group (5)
		Remote learning (2)
		Teenage year specifics (1)
		Playing in musical concerts (1)
		Lack of social environment (1)
	Lack of parental support	Excessive strain on parents (1)
Individual differences	Health issues	Specific health issues (8)
	Learning difficulties	Specific learning difficulties (3)
	Instrument preference	Changing a musical instrument (2)
	Stage fright	Student’s stage fright (2)

**Table 3:** External factors.

*Theme*	*Category*	*Code*
Teacher’s approach	Adverse teacher-student relationship	Negative teacher-student relationship (6)
		Positive teacher-student relationship (3)
	Insufficient student-centered teaching	Teacher’s competence in interpersonal relations (6)
		Individualized teaching (2)
	Inadequate teacher-parent relationship	Inadequate teacher-parent relationship (1)
Social environment	Unstimulative classroom atmosphere	Negative student-teacher relationship (6)
	Limited social interaction opportunities	Playing in musical group (5)
		Remote learning (3)
		Lack of concerts (2)
		Digital age (1)
Music school’s curriculum	Music theory and solfége	Music theory and solfège (18)
	Unappealing musical repertoire	More demanding repertoire in the higher classes (7)
		Repertoire for technique development (3)
		Musical pieces from popular music genres (3)
	(Over)emphasis on musical literacy	Reading music (4)
Resources	Limited financial resources	Limited financial resources (2)
		Excessive strain on parents (1)
	Limited musical instrument access	Limited musical instrument access (1)
Workload	Competing extracurricular commitments	Competing extracurricular commitments (23)
	Raising academic demands in general education	Raising academic demands in general education (1)
	Excessive repertoire load	Excessive repertoire load (2)
	Pressure of musical instrument assessments	Pressure of musical instrument assessments (2)
	Parental support strain and logistics burden	Excessive strain on parents (1)

**Autonomy** is affected when students engage in music education not out of personal interest but due to parental influence. The code *Learning music for parents’ sake* indicates that at least two instances were noted where students’ participation was more about fulfilling parental expectations rather than their own choice. One student said: “M*y mother persuaded me to give it a year [..]. She tried to convince me for another year, but I said no.”* This lack of personal choice is further complicated by a restrictive approach to learning, such as a limited repertoire where a student notes the gap between what is taught and personal interests, outlined by the instrument teacher: *“We have a gap between what is taught in music school and what they would like to learn.”* Students seek connections between their music education and the music they encounter daily, indicating a desire for relevant and relatable learning content. Several instrument teachers emphasized the problem of repertoire for technique development, with one of them stating: *“Students like scales and etudes the least.”* While necessary for skill advancement, focusing solely on technique can limit students’ creative expression and choice, impacting their autonomy. One student pointed out that his dream music school would be the place where “*you could write your own notes with a magic wand.”* Several teachers and parents pointed out the importance of teachers’ flexibility, with one of the instrument teachers stating: *“It seems to me that we have quite a big role to play. I know there have been many cases where I feel that the teachers have been a little bit too insistent in trying to accommodate the child, and that is because it is easier to get away with someone diligent, someone who practices, someone who is talented. Because, if you have an untalented child, who does not work, who is a little bit problematic, […] it takes a lot of your energy as a teacher, a lot of knowledge, a lot of patience, to get to a certain result, compared to having a student who you just tell to learn something at home, and he does it.”*

**Competency** concerns arise when students experience setbacks in competitions or perceive their abilities as inadequate. Musical competitions can be disheartening, especially if students feel they have failed to meet expectations, which can undermine their sense of competence, as noted by the instrument teacher’s comment: “*One drop-out student was disappointed after the competition because expectations were higher, and he was very upset afterward.”* Furthermore, exams can also be a source of stress and may contribute to students’ perceptions of diminished competence if they do not perform well. One of the instrument teachers highlighted that *“music school is demanding; that’s a fact; it takes a lot of perseverance and patience for a very small result.”* Another instrumental teacher pointed out: “*Maybe when that leap happens, say in third, fourth grade, it gets harder and harder, then maybe they see that they just cannot handle the repertoire anymore.*” When asked what it would be that would make you like music school more to continue, the student said: *“Maybe the notes.”* One of the instrument teachers emphasized: *“I would put exams first [in terms of what they do not like]. They are a bit afraid of them, I would say.”* Students might perceive their abilities as inferior, which can discourage continued participation in music education. Comparisons to siblings who may be more musically inclined can create feelings of inadequacy. One parent described: *“So, obviously, she’s more of a competitive type of person, and basically, at the beginning, she wanted to compare with her sister a little bit because the sister started going to music school.”* As students progress, increased difficulty may exacerbate feelings of incompetence, particularly if they are not adequately prepared, indicating that the perception of effort versus reward may influence their decision to continue. *Teacher: “I know of cases where students went to music school and then found out that they really had no [musical] talent.”*

In terms of **relatedness**, poor teacher-student relationships, limited peer relations, and lack of parental support were identified as key categories. Teacher-student relationship quality indicates the importance of positive relationships for student retention, where a lack of quality interaction can lead to dropout. Inflexible or impatient teaching can damage the relational bond necessary for student motivation, as outlined by a parent: *“She expected a bit more socializing, a bit more singing together. There was none of that.”* Poor teacher-student relationships reinforce the teacher-student dynamic’s impact, with poor relationships contributing significantly to dropout rates. One parent said: *“I know that this relationship with the teacher is very important; to feel accepted.”* Parental support is also a key factor, as noted by the instrument teacher: *“Parents need to encourage and support students in a way that is kind to them.”* Group playing can enhance a sense of belonging, but negative experiences within these groups can also deter students. One of the parents pointed out: *“In fact, she was so bored that she would rather go dancing somewhere because there are other children there.”* Instrumental teacher emphasized students’ affinity for music-making with others: *“I think that, at least for my instrument, they like chamber music and orchestra the best*.” Performance opportunities can strengthen relatedness, though, for some, it may also be a source of anxiety or a feeling of exclusion if not handled well. One of the instrument teachers emphasized: *“They feel good after a successful performance.”*

Lastly, **students’ individual characteristics**, such as stage fright, musical instrument changes, and specific learning difficulties, point to personal challenges affecting their learning experience. One instrument teacher noted that “s*ome students have such a stage fright that they see they will not get through it and quit rather than suffer because they know what’s coming.”* A parent commented, “*Because she was sick a lot, she was at home often. [..] Half the days she was sick, half the days she was at school. [..] So we said, let us leave it.”* On the other hand, another instrument teacher commented, *“Sometimes they might find that they have picked the wrong instrument [..]. We have quite a few cases where they dropped out, and then they realized they had chosen the wrong instrument.”* These categories highlight the profound effect of individual psychological factors on the learning process.

### External factors

External factors focus on elements outside the student’s immediate control, including teaching approaches, the social environment, curriculum content, resource availability, and overall workload.

The interpretation of external factors related to dropout from music schools in our sample reveals that the **teaching approach**, including the quality of teacher-parent and teacher-student relationships, significantly impacts student retention. Inadequate teacher-parent relationships can lead to misunderstandings or a lack of support for the student’s musical journey, as a teacher indicated: *“Knowing to raise musical kids having no musical instrument at home.”* This underscores the importance of a supportive network extending beyond the classroom. Adverse teacher-student relationships and insufficient student-centered teaching were also mentioned, highlighting the need for teachers to connect with students individually. One parent explained his view on his daughter’s dropout from music school: *“Looking at my daughter when she stopped piano lessons after the third grade, it was mainly the relationship between the individual teacher and her that was to blame.”*

Similarly, the **social environment** within schools is crucial, as a parent’s remark, *“There was no socializing, so she found it boring,”* reflects the need for a more engaging and interactive learning atmosphere, with the classroom dynamic crucial for maintaining student interest. The limited social interaction and opportunities, exacerbated by remote learning, challenge student engagement and enjoyment. One parent mentioned the lack of concerts as one of the factors for dropping out of music school: *“There were no concerts during the quarantine. There was no socializing.”*

Additionally, the **music school’s curriculum** can serve as a deterrent if it does not align with students’ interests or engage them creatively. An unappealing musical repertoire and overemphasizing musical literacy, such as music theory and solfège, rather than performance and creativity, can dampen enthusiasm. An instrument teacher highlighted musical theory and solfeggio as an important factor for dropping out of music education: *“My pupils have a thing against it. When moving from sixth to seventh grade, many would continue to learn a musical instrument, but they do not want to learn solfeggio anymore.”* A music teacher commented, *“Repertoire should be different, not just a part of the music to be played [..] of course, is sort of compulsory.”* Such feedback suggests that a one-size-fits-all curriculum may not adequately serve students’ diverse needs and interests.

Resource limitations, particularly financial constraints and lack of instrument access, also emerge as significant barriers. Teachers observe the difficulty in *“raising musical kids having no musical instrument at home,”* emphasizing the need for accessible resources for students to practice and improve.

Furthermore, the **workload** from the music school and general education demands can overwhelm students, as indicated by a teacher who notes the burden of *“music education [..] and they do have a lot at school.”* The compounded pressures of academic and musical assessments and an excessive repertoire load may lead to stress and disengagement among students. One of the instrument teachers emphasized: *“There are lots of activities. Apart from primary school, music school is not the only activity in the afternoons, but there is one sport, another sport, there is a club, there are foreign languages, there is computing, there are class activities. It is exhausting, and then one thing has to fall away. Typically, this is the music school, which requires significant effort and time.”*

## Discussion

The primary objective of our research was to examine the contributing factors behind dropout rates and decreased motivation among music students aged 7 to 15 attending public music schools in Slovenia. No empirical study has been conducted to explore the key reasons for this phenomenon in Slovenia. Therefore, our study tried to bridge this gap. Furthermore, we aimed to explore the intertwining of external and internal factors that lead to dropouts from the perspective of students, their parents, music instrument teachers, music theory teachers, and school principals.

The discussion is structured around nine themes that emerged from thematic analysis, categorized into four internal and five external factors.

Among the **internal factors**, three align with the principles of self-determination theory (SDT) proposed by Deci and Ryan (1985). The most perceived factor for dropout was the lack of perceived autonomy, followed by the absence of feelings of competence and deficiency in relatedness. Additionally, individual differences such as health issues, learning difficulties, instrument preference, and stage fright emerged as the fourth internal factor influencing dropouts.

Several previous studies in the field of instrumental music education have drawn upon SDT to understand motivational dynamics (Evans et al., 2012; Evans, 2015; Freer and Evans, 2018; Gerelus et al., 2020; Lee and Leung, 2020; Oliveira et al., 2021; Shaheen, 2022). Evans (2015) highlighted the importance of situating music learning within a social context that fulfills fundamental psychological needs—competence, autonomy, and relatedness—closely linked to musical engagement and overall well-being. When students lack intrinsic motivation and the learning environment fails to support these psychological needs, dropout risk significantly increases (Evans et al., 2012). Drawing insights from SDT, music educators can promote autonomous integration, leading to improved academic achievement and reduced dropout rates (Gerelus et al., 2020).

***Autonomy*** emerged as a crucial internal factor influencing dropout rates, particularly when students engage in music education under parental influence rather than personal interest. Our results align with King’s study (2016) and the research findings of Gerelus et al. (2020), which confirmed that dropout music students are significantly less autonomously motivated than their persisting peers. Interestingly, parental over-involvement in their child’s music lesson setting may contribute to a lack of autonomy (Gerelus et al., 2020), especially if instrument learning is parent-initiated, as reported in our results. Parent-initiated music learning, which can result in parental pressure on children, may lead to increased stress levels in children learning music, ultimately hindering their progress and enjoyment of the activity (McPherson et al., 2012). The key lies in finding the right balance between encouraging a child’s learning of musical instruments—where active parental involvement is crucial for a child’s success in the musical domain—and simultaneously allowing the child autonomy as a parent without exerting excessive control. Autonomy is particularly important because it affects students’ self-efficacy and well-being. McPherson and McCormick (2006) found that self-regulation, which includes aspects of autonomy, positively influences self-efficacy in young musicians. Another study by Creech and Hallam (2011) explored the role of autonomy-supportive teaching practices in enhancing self-efficacy beliefs in music students. They found that teachers who encouraged students to take ownership of their learning process and provided opportunities for self-directed practice and exploration fostered higher levels of self-efficacy among their students.

When students feel empowered to make choices about their musical learning and expression, it can positively impact their overall well-being. For example, a Bonneville-Roussy et al. (2020) study reported that teachers’ autonomy-supportive behaviors were related to students’ well-being, whereas controlling behaviors hindered well-being. The lack of autonomy is further exacerbated by limited repertoire choices and a perceived gap between taught content and personal musical preferences. Repertoire is the most motivational tool for starting to play an instrument, and the pieces play a significant role in progress (Feschanka, 2021). Therefore, one of the main motivational strategies in instrumental teaching is to provide music students with the autonomy to select between several musical pieces.

Teachers’ lack of flexibility was reported to be an important demotivator in the music learning process. The desire for relevant and relatable learning content is evident among students, emphasizing the importance of aligning the curriculum with their interests for enhanced engagement. As outlined by Šimunovič and Habe (2024), musical genre diversity contributes to the motivation of young musicians highlighting the need for supportive environments that foster positive self-perception and motivation. The latter is aligned with the research findings of Bernabé-Valero et al. (2019), which highlight the importance of effort in sustaining motivation in music students, including perceptions about one’s own skills, satisfaction with achievements, effort, the importance of music in one’s life, and perception of the sacrifice made.

***Competency*** concerns stemming from setbacks in competitions, exams, and comparisons with more musically inclined siblings contribute to diminished competence perceptions. The perception of underdeveloped musical abilities and skills among music students highlights the multifaceted nature of competency in music education. Our study identifies the challenge posed by a more demanding repertoire in higher classes. This aligns with previous research indicating that the complexity and difficulty of musical pieces can impact students’ perceptions of their own abilities (Sosniak, 1985). Issues such as difficulties in reading music and performance anxiety during musical exams that emerged in our study can undermine students’ confidence in their musical abilities (McPherson and McCormick, 2006). Several previous studies involved competency in exploring (a)motivation in music activities engagement (Costa-Giomi, 2004; Costa-Giomi et al., 2005; StGeorge, 2006; King, 2016; Gerelus et al., 2017, 2020). The previous findings suggest that the lack of competency, such as less overall musical ability and musical achievement (Gerelus et al., 2017, 2020), contributes significantly to the attrition from music schooling. The perception of effort versus reward plays a role in students’ decisions to continue.

***Relatedness*** factors, encompassing poor teacher-student relationships, limited peer interactions, and lack of parental support, significantly impact dropout rates. As prior studies have indicated, a psychological requirement crucial for individuals’ continued engagement with music is the need for relatedness (Oliveira et al., 2021). Music facilitates establishing social connections among individuals with similar musical interests (McPherson et al., 2012). Previous research confirms that satisfaction of the need for relatedness is one of the most significant factors influencing the quality of motivation in the music school context (Evans et al., 2012; Tucker, 2020; de Bruin, 2021).

Our results reveal that positive teacher-student relationships and parental encouragement emerge as essential for student retention. These findings corroborate with Creech and Hallam (2011), who investigated the influence of student-teacher and student-parent dynamics on various aspects such as self-esteem, self-efficacy, motivation, enjoyment of music, musical achievement, and satisfaction with lessons. Their findings revealed that reluctance in student-teacher interaction negatively impacts several motivational factors, including enjoyment of music, satisfaction with music lessons, motivation, and self-esteem. Moreover, a positive student-teacher relationship can also foster or promote autonomy (Küpers et al., 2014; Comeau et al., 2015). Regarding the importance of parental support, previous findings suggest that openness to parental support demonstrates a positive correlation with all motivational aspects (enjoyment of music, satisfaction with instrumental lessons, motivation, self-efficacy, and self-esteem), except for musical achievement, suggesting a potential hindrance to musical progress through parental support.

Group activities and performance opportunities can strengthen the sense of relatedness. However, negative experiences with them may lead to anxiety or feelings of exclusion. Burnard and Dragovic (2015) outlined that collaborative creativity in instrumental group music learning is a site for enhancing music students’ well-being. The thrill of performing in front of an audience, especially when performing in a group, can help maintain the motivation to engage in music performance activities (Lowe, 2012). Anticipated public performances are strong incentives for musicians to engage in practice (Hallam, 1997; Woody, 2001; Burwell and Shipton, 2011). However, performing solo can also be a huge demotivator and sometimes even a reason for attrition from a music school. Additionally, if a music student does not feel connected with the group he is performing with or does not share performing goals with the other group members, he can experience significant frustration and distress.

***Students’ individual differences***, such as stage fright, instrument preference changes, health issues, and learning difficulties, underscore the profound influence of personal challenges on the learning process. These factors highlight the importance of recognizing and addressing individual needs to support students effectively. Stage fright is reported to be one of the main stressors in the life of young musicians. Research suggests that high levels of music performance anxiety can lead to decreased motivation and ultimately contribute to dropout rates among music students (Habe and Kržič, 2017). Factors such as fear of judgment, self-doubt, and pressure to perform flawlessly can exacerbate performance anxiety, leading to negative outcomes in music education. Understanding and addressing the root causes of music performance anxiety are crucial for creating supportive learning environments that foster students’ long-term engagement and success in music education.

**External factors**, including the teacher’s approach, social environment, music school curriculum, resources, and workload, also contribute to dropout rates. Inadequate teacher-parent relationships and adverse teacher-student relationships emphasize the need for supportive networks beyond the classroom. The social environment, curriculum alignment with students’ interests, and accessible resources play pivotal roles in student engagement.

***An unsupportive teacher’s approach***, including inadequate teacher-parent relationships, adverse teacher-student relationships, and insufficient student-centered teaching, emerged as one of the reasons for attrition. Particularly negative teacher-student relationships and a lack of teachers’ competence in interpersonal relations were highlighted. As Hansen and Imse (2016) stated, student-centered teaching practices incentivize 21st-century skills in music education. With the teacher as facilitator, young musicians are prompted to self-reflect, evaluate their peers, and problem-solve regarding music-making and creation.

A ***non-stimulative*** classroom atmosphere and limited social interaction opportunities emerged as categories regarding the ***social environment***. In our opinion, the latter still reflects the consequences of the coronavirus pandemic (Šimunovič, 2020), when remote learning, lack of concerts, and the absence of group play were crucial reasons for music pupils’ demotivation.

The greatest reported challenges regarding the ***music school curriculum*** are music theory and solfege. Many young pupils struggle with these subjects and fail to see their practical value. Therefore, cross-curricular connections in music schools are very important (Gruden, 2019). Unappealing music repertoire, including pieces for technique development, a lack of musical pieces from popular music genres, and especially highly demanding repertoire, was also reported as a reason for music pupils’ attrition. Lowe (2012) reports that music pupils in their study expressed a preference for regularly changing repertoire, favoring faster, rhythmic, and memorable music. Thus, instrument instructors are urged to select repertoire with these motivational attributes and regularly vary the repertoire to sustain student interest when feasible. Additionally, providing students with some degree of choice in repertoire may foster feelings of autonomy and independence.

***Resource limitations***, particularly financial constraints and lack of instrument access emerge as significant barriers to music education. The study emphasizes the need for accessible resources to ensure students can practice and improve effectively. Our results align with previous findings reporting socioeconomic status as an important predictor of motivation for engaging in music activities (Hoffman, 2013; Jeppsson and Lindgren, 2018).

The ***workload from music schools and general education demands*** presents a potential source of stress and disengagement. Balancing academic and musical assessments and an excessive repertoire load requires careful consideration to prevent overwhelming students. Riley (2016) reports that academic overload and extracurricular overload can contribute to burnout in young musicians.

Based on the formulated themes categorized into internal and external factors, we can observe that many themes intertwine and can be found among both sets of factors. This can be observed in themes such as music repertoire, which emerged in internal factors under lack of autonomy, and external factors under music schools’ curriculum. Additionally, the teacher-student relationship emerged as an internal factor under lack of relatedness and as an external factor under the teacher’s approach. The intertwining could also be observed between the social environment, a theme that emerged in external factors, and relatedness, formed as an internal factors theme.

As revealed in our thematic analysis, the intertwining of internal and external factors is consistent with previous research findings, which have shown that internal and external factors are constantly connected and influence each other (Rotar Pance, 2006).

## Conclusion

In conclusion, our study provides valuable insights into the multifaceted factors contributing to dropout rates in Slovene public music schools. The findings underscore the need for a holistic approach, addressing both internal and external elements, to create supportive environments that foster autonomy, competency, and relatedness and accommodate individual characteristics. As Alessandri et al. (2020) suggest, institutions need to embed health and well-being into a “living curriculum” to accommodate the needs of different students. Implementing these insights may contribute to reducing dropout rates and enhancing students’ overall music education experience.

Lastly, it is important to highlight that this is the first systematic empirical study in Slovenian music education focusing on the current challenges of finding solutions to maintain learning motivation and reduce dropout rates in the music school environment. The study will serve as a foundation for conducting quantitative research, the results of which will provide a clearer picture of the vision for the future development of Slovenian music education.

By addressing the factors identified in our study, particularly those rooted in SDT, music educators and policymakers can design interventions and create environments that foster students’ autonomy, competence, and relatedness. This holistic approach is crucial for nurturing intrinsic motivation, enhancing musical engagement, and ultimately reducing dropout rates in music education settings.

## Data availability statement

The raw data supporting the conclusions of this article will be made available by the authors, without undue reservation.

## Ethics statement

The studies involving humans were approved by University of Maribor’s Faculty of Arts Ethics Committee. The studies were conducted in accordance with the local legislation and institutional requirements. Written informed consent for participation in this study was provided by the participants’ legal guardians/next of kin.

## Author contributions

AK: Writing – original draft, Writing – review & editing. KH: Writing – original draft, Writing – review & editing. BR: Writing – original draft, Writing – review & editing. ML: Writing – original draft, Writing – review & editing.
